# Mesoglycan for the secondary prevention of superficial vein thrombosis: a randomized, controlled, double-blind study (METRO Study)—rationale and protocol

**DOI:** 10.1007/s11239-023-02896-6

**Published:** 2023-11-06

**Authors:** G. Camporese, E. Bernardi, C. Bortoluzzi, F. Noventa, P. Simioni, for the METRO Investigator Study Group

**Affiliations:** 1 Department of Medicine, University of Padua, Padua, Italy; 2 Thrombotic, and Hemorrhagic Disorders Unit, Department of Systems Medicine, University - Hospital Padua, Padua, Italy; 3 Emergency Room, Department of Emergency and Accident Medicine, San Camillo Hospital, Treviso, Italy; 4 Division of Internal Medicine, Department of Internal Medicine, Santa Maria delle Grazie Venice Civil Hospital, Venice, Italy; 5 QUOVADIS Association and Department of Molecular Medicine, Padua University Hospital, Padua, Italy

**Keywords:** Superficial vein thrombosis, Secondary prevention, Treatment, Glycosaminoglycans

## Abstract

No data is available about pharmacological secondary prevention of superficial vein thrombosis (SVT) despite 10–15% of patients develop venous thromboembolic complications at 3–6 months after an adequate treatment of the acute phase. To verify efficacy and safety of mesoglycan in secondary prevention of SVT recurrence and venous thromboembolic complications. Phase III multicenter, double-blind, randomized, superiority trial comparing mesoglycan 50 mg bid vs placebo in consecutive patients with a SVT extended at least 5 cm, after the initial 45-day treatment course with fondaparinux 2.5 mg once-daily. Primary efficacy outcome: SVT recurrence/extension, symptomatic venous thromboembolism (VTE), asymptomatic proximal deep-vein thrombosis, death. Primary safety outcome: major bleeding. We hypothesized a 12-month 15% incidence of the primary efficacy outcome in placebo group and a 50% risk reduction in mesoglycan group. A bilateral log-rank test with a sample of 650 patients (randomization 1:1) reach a 90% power, with an α-error of 0.025, of detecting a 7.0% difference (HR = 0.51) after 12 months of treatment, considering a 10% patients drop-out. At deadline (December 31, 2022) 570 patients have been randomized (10% drop rate). Mean age was 63.9 years, 58.8% were women. SVT involved great saphenous vein in 69.3%, small saphenous vein in 13.1%, and collaterals in 17.6% of patients. SVT was the first event in 61.7%, a recurrence in 38.3%, provoked in 50.2% and unprovoked in 49.8%. Patients not experiencing a primary outcome, or not retiring their consent will be followed up to December 31, 2024 when the final data analysis will be performed

ClinicalTrials.gov: NCT03428711.

## Highlights

- Superficial vein thrombosis is frequently associated to venous thromboembolism
- No data is available about secondary prevention of superficial vein thrombosis
- Mesoglycan could prevent recurrences with a long-term treatment versus placebo
- Lacking data on the natural history of the superficial vein thrombosis will be recorded

## Introduction

Superficial vein thrombosis (SVT) is a relatively common clinical condition characterized by a combination of thrombosis, inflammation, and pain along the course of a superficial vein [1]. A recent epidemiological community study clearly showed an incidence of SVT of 0.64% per year, which is six times higher than the incidence of venous thromboembolism (VTE), with an annual diagnosis rate ranging from 0.04 [95% CI, 0.00–0.10%] in men aged 18–39 years to 2.2% [95% CI,1.59–2.78%] in women aged > 75 years [2].

Considered a benign disease for a long time, SVT was recently shown to be associated with deep vein thrombosis (DVT) and symptomatic pulmonary embolism (PE) in up to 25% and 13% of cases, respectively [2, 9]. Proximal SVT (thigh) was also found to be associated with asymptomatic PE in up to 33% of cases [2, 9].

Patients with SVT and obesity, cancer, male sex, personal or family history of VTE, or severe chronic venous insufficiency are at increased risk of developing short- and long-term complications of VTE [10–14].

Indeed, recognizing a strong association between SVT and VTE in the last 10–15 years significantly modified the perceived risk of the former and its therapeutic approach, especially in the acute phase. In this respect, only fondaparinux (2.5 mg OD for 45 days) was shown to significantly reduce VTE complications, SVT extension, or recurrence by approximately 80% compared to placebo; while studies with low molecular weight heparins gave conflicting or less conclusive results [9, 10, 15, 16]. Consequently, fondaparinux (2.5 mg OD for 45 days) currently represents the first choice for the acute phase treatment of patients with SVT of the lower extremities [17].

Once completed the treatment of the acute phase, several studies have shown the utility of a treatment extension, either with vitamin K antagonists, or with direct oral anticoagulants, or low-molecular-weight heparins, or even aspirin or sulodexide as compared to no treatment in preventing VTE recurrence in patients with a first DVT and/or PE event [18–22].

On the contrary, no data is available on the need for extended treatment / secondary prevention of thromboembolism after SVT. However, despite adequate anticoagulant treatment in the acute phase, the incidence of VTE complications is as high as 15% at 3–6 months from the index event [3, 5, 15].

Mesoglycan is a complex mucopolysaccharide formed by a blend of natural glycosaminoglycans, mainly heparin-sulfate and dermatan-sulfate. Pharmacokinetic animal studies (in rats and monkeys) with tritium-labeled mesoglycan showed peak plasma levels of mesoglycan between 30 and 120 min after oral administration [23, 24]. The steady-state condition was maintained for up to 7 h; presumably due to a slow-release of the drug, after absorption by the gut-wall [23, 24]. To the contrary, peak plasma levels were recorded almost instantaneously after intravenous administration, with a subsequent rapid concentration fall within 60 min, following a biphasic course [23, 24].

The pharmacological activity of mesoglycan is expressed at the endothelial and subendothelial level, and is mainly due to the presence of heparan and dermatan sulfate, which are physiological constituents of the vessel wall.

Concerning its pharmacological effects, mesoglycan is a mild thrombin inhibitor with complementary action mediated by antithrombin and heparin-cofactor II, and is also profibrinolytic, stimulating the tissue plasminogen activator. It also exerts an antiatherogenic effect, via the inhibition of platelet adhesion, the stimulation of enzyme lipoprotein lipase, and the inhibition of the proliferation of smooth muscle cells of the tunica media; and an antiinflammatory effect via reduction of plasmatic concentration of several different cytokynes (such as MMP2, MMP9, IL6, TNFα, ICAM1, VCAM1) [25–31]. Furthermore, limited to the venous side of the circulatory system, mesoglycan is able to restore the physiological properties of the selective barrier represented by capillary endothelium, restoring flow-mediated vasodilation in subjects with endothelial dysfunction, repairing venous wall damage caused by chronic venous insufficiency, and accelerating the healing of venous ulcers [25–28]. Mesoglycan does not significantly influence coagulation parameters. In the literature there is only a small study showing a statistically significant decrease in fibrinogen plasma concentrations and an improvement in erythrocytes filterability following mesoglycan treatment, but no effect was found on prothrombin time, activated partial thromboplastin time or antithrombin levels [32].

While the efficacy of mesoglycan in preventing recurrence after a (first) episode of DVT is documented, there is no published evidence on the efficacy and safety of mesoglycan for the prevention of recurrence in patients with SVT of the lower limbs [33, 34].

### Aim of the study

The aim of the METRO (Mesoglycan for secondary prevention of superficial vein thrombosis), a randomized controlled and double-blind study, is to evaluate the usefulness of mesoglycan for the secondary prevention of VTE, after the initial 45-day course of subcutaneous fondaparinux (2.5 mg OD), in patients with SVT of the lower limbs.

## Materials and methods

### Design of the study

The METRO study is a multicenter, phase 2, randomized, double-blind, superiority trial that compares mesoglycan 50 mg BID with a matching placebo. Using a placebo as a comparator is justified by the absence of scientific evidence on the efficacy and safety of any treatment in this setting. The METRO study is conducted in 19 Italian Units of Vascular Medicine, all with significant experience in the management of VTE. The Coordinating Centre will be the General Medicine Unit and the Thrombotic and Haemorrhagic Disorders Unit at the Department of Medicine—Padua University Hospital, Italy. The study protocol has been reviewed and approved by the Ethics Committee of the Padua University Hospital, Italy, and by those of each participating center.

Before inclusion, patients/participants are requested to sign a written informed consent form.

An independent data safety monitoring board (DSMB), blinded to the patient's treatment allocation, formed by three highly qualified clinicians in the field of VTE, will adjudicate all efficacy events, monitor patients' safety, and vouch for the data quality.

### Study population and randomization

Subjects of both sexes, > 18 years of age, with objectively documented SVT of the lower limbs, initially extended for at least 5 cm, who had completed the standard 45-day course with fondaparinux 2.5 mg OD, were asked to participate in the study. Patients could only be included if they consented to participate within two weeks from treatment withdrawal. The complete list of inclusion and exclusion criteria is reported in Table 1.

**Table 1:** Inclusion and exclusion criteria for METRO study

Inclusion criteria	Exclusion criteria
Age ≥ 18 years Objective diagnosis of SVT of the lower limbs documented with Color Coded Doppler Ultrasound of at least 5 cm extension, not involving the last 3 cm of the great saphenous vein and of the small saphenous vein, close to the respective sapheno-femoral and sapheno-popliteal junction Completion of the initial treatment of the acute phase with fondaparinux 2.5 mg once daily for 45 days With a negative ultrasound scan for SVT recurrence/extension and/or DVT at the end of the acute phase treatment. treatment	Little adherence to the treatment of the acute phase with Fondaparinux or its premature withdrawal before the deadline of 45 days Interval between the end of fondaparinux treatment and recruitment > 15 days Mandatory anticoagulation for other clinical diseases than SVT Life expectancy < 24 months Scarce compliance or impossibility of filling or answering the questionnaires Pregnancy or breastfeeding Severe muscoloskeletal disability or prolonged immobilization Concomitant participation in another clinical trial or previous participation in the last 3 months Overt post-thrombotic syndrome with a Villalta Score > 4 Chronic lymphedema of the lower limbs Recent (< 3 months) or planned surgery for varicose veins or PTA angioplasty Dyalisis Malabsorption or malnutrition Chronic use of high doses of phlebotonics, steroids, or nonsteroidal anti-inflammatory drugs, mandatory dual antiplatelet therapy or aspirin > 160 mg once daily, central painkillers Known hypersensitivity to mesoglycan or heparin or heparinoids Intolerance to galactose or lactase enzyme deficiency Bleeding tendency

After signing the informed consent, eligible subjects will complete the screening visit. Only patients who meet the inclusion and exclusion criteria will be included.

The included patient will first undergo bilateral color-coded Doppler ultrasound (CCDU) of the lower limbs to exclude concomitant involvement of the deep venous system or an extension of the index SVT. Furthermore, blood sampling for alanine-aminotransferase, aspartate-aminotransferase, creatinine, complete blood count, prothrombin time, activated partial thromboplastin time, and C-reactive protein will be performed.

A computer-based centralized randomization, with 1:1 allocation between mesoglycan and placebo, and random variable size blocks (4 to 6 units), stratified for age (< 60 y, >  = 60 y), and gender (F/M), will be run through a dedicated application (REDCAp). Patient recruitment is expected to be completed in 24 months.

### Treatment

Patients will receive a hard capsule of 50 mg mesoglycan or a matching placebo twice daily for 12 months. Treatment packages will be labeled with a bare numerical code without reference to the content. The match between the numerical code and the package content will only be known to the packaging manager. Patients, investigators, the steering committee, and DMSC will be blinded to treatment group allocation. The blinding seal will only be opened after the database lock to allow the analysis of the study results, except for in the case of a medical emergency, for which it is considered essential to know the type of treatment.

Each participating center will keep a printed drug accountability form for each patient included in the study (drug amount delivered, consumed or returned).

### Patient flow

Medical control visits will be scheduled 3, 6, 9, and 12 months after randomization for every included patient, during which a complete evaluation of their clinical status, compliance with treatment, concomitant treatments and adverse events will be performed. At each visit, patients will also receive the revised Venous Clinical Severity Score (rVCSS), which accounts for changes in disease severity over time and in response to treatment, and the VEINES/Sym-QoL questionnaire, a disease-specific quality of life instrument for chronic venous disorders of the leg [34–39]. A CCDU examination and a blood sample will also be scheduled for the 12 month visit.

At the end of the 12-month treatment cycle, patients will enter another 12-month clinical follow-up period, during which they should not receive any experimental treatment; for a total study time of 24 months. A final visit will be scheduled 24 months after randomization in patients who complete the study course.

**Fig. 1:**
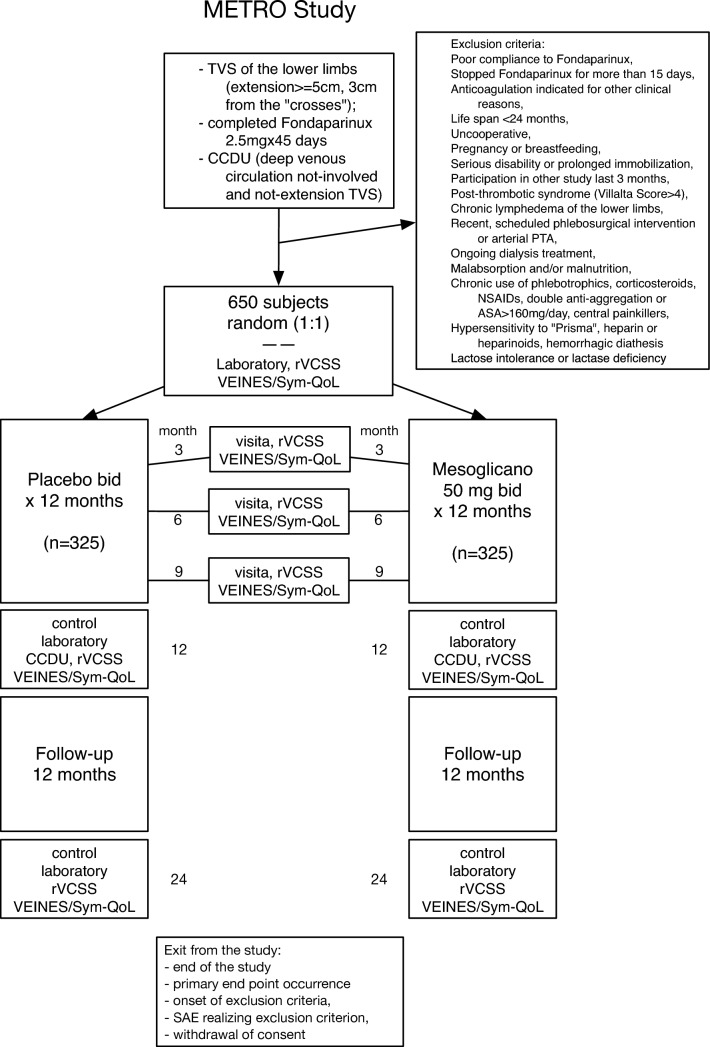
Flow of the patients through the study

Patients who meet exclusion criteria at any time during the study, become pregnant, do not comply with study purposes or withdraw their consent will be censored. Patients who withdraw experimental treatment will continue to observe according to the study protocol until the end of the study. The planned flow of the patient is shown in Fig. 1.

### Study outcomes

The *primary efficacy outcome* of the study will be an objectively documented symptomatic recurrence or extension of SVT, symptomatic DVT (proximal or distal), asymptomatic proximal DVT, symptomatic pulmonary embolism (fatal / nonfatal), or death, whichever comes first, during the treatment period.

The *secondary efficacy outcome* will be: the occurrence of asymptomatic recurrence, extension, or asymptomatic distal DVT; the rVCSS score and the VEINES / Sym-QoL questionnaire; the impact of elastic compression therapy on SVT treatment; the association between CRP levels and primary efficacy outcomes; the natural history of SVT in patients allocated to placebo; the evaluation of the prolonged protective effect of mesoglycan after the first 12 months of treatment.

The *safety outcome* will be the incidence of major bleeding, as defined by the International Society on Thrombosis and Hemostasis (ISTH) criteria: fatal and/or symptomatic bleeding in a critical area or organ (e.g., intracranial, intraspinal, intraocular, retroperitoneal, intraarticular, pericardial, intramuscular with the associated compartmental syndrome); and/or in the case of clinically overt bleeding associated with a hemoglobin decrease of at least 20 g/L or requiring transfusion of 2 or more units of packed red blood cells [40].

### Outcome assessment

Patients will receive detailed instructions on how to contact their referral center if symptoms suggestive of efficacy or safety events occur during the course of the study (treatment period and clinical follow-up).

Patients with suspected recurrent SVT or DVT will undergo CCDU. As previously described, whole-leg CCDU will be performed according to a standardized protocol [41]. Briefly, the proximal venous system of the lower limbs (common, superficial, and deep femoral veins, and popliteal vein to the trifurcation) is examined first with the patient lying supine; then the distal venous network (tibio-peroneal and muscular veins) is scanned, with the patient sitting. We do not routinely investigate the anterior tibial veins. Therefore, vein incompressibility is the only diagnostic criterion used to rule in proximal and/or distal DVT and SVT. Patients with symptoms suggestive of PE are investigated with Computed Tomography Pulmonary Angiography (CTPA) or Pulmonary Ventilation (V)and Perfusion (Q) scan. PE is adjudicated in the presence of an intraluminal filling defect involving the principal, lobar, or segmental branches of the pulmonary artery. If the patient dies, the cause of death is adjudicated as "likely" or "unlikely" due to PE based on autopsy findings, if available, or on clinical grounds.

### Data Collection

Data will be recorded on an “Electronic Data Capture” (EDC) system, based on the “Research Electronic Data Capture” online platform (REDCap, produced and distributed by Vanderbilt University and “REDCap Consortium”), in compliance with article n. 13 of the GDPR (EU 2016/769) [42].

The personal data of the subjects enrolled will be treated with maximum confidentiality, according to the terms of Italian law (DL 211/2003 and subsequent amendments and additions) and GDPR [Regulation (EU) 2016/679 of the European Parliament and of the Council of 27 April 2016].

In particular, the subject's personal data will be known only to the principal investigator and his collaborators who follow the patient and collect the treatment consent. Each enrolled subject will be distinguished only by a unique numeric identifier. The use and maintenance of the REDCap platform are guaranteed by an administrator who manages the user's privileges with a flexible and granular authorization system. REDCap applies the permissions granted to each user who connects via a web browser and the SSL encryption protocol. REDCap allows a complete "audit" of the procedures performed by each user by logging all operations on the data, including viewing and exporting. The operational control log stores the date, time, and operating behavior of the user, allowing a complete review and remote monitoring of the clinical study if necessary. All users accessing the EDC platform (the investigators, the members of their staff, and the staff of the Promoter) must complete a training event to increase the reliability, quality, and integrity of the data recorded in the EDC platform. REDCap implementations allow compliance with the most common industry standards and EMA requirements (GCP, Privacy-IT: DL 211/2003 and subsequent amendments and additions, GDPR, and FDA (21-CFR2-Part 11).

### Statistical analysis and sample size

From the available data in the literature we hypothesize a risk of the occurrence of the primary efficacy outcome in the placebo group at the end of the treatment of 15% (that is, a cumulative proportion of 85% of subjects without events) and a 50% decrease in risk in the mesoglycan group (HR = 0.5) [3, 5, 9].

A two-sided log-rank test with an overall sample size of 650 subjects (of which 325 are in the placebo reference group and 325 in the mesoglycan treatment group) achieves a power of 90%, at a significance level (alpha) of 0.025, to identify a difference of 7.2%, between 15.0 and 7.8% (the proportions of subjects with primary endpoint in the placebo and mesoglycan group, respectively) after 12 months of treatment. This corresponds to a hazard ratio (HR) = 0.50. Subjects will be enrolled in the study for 24 months, and 50% of enrollments will be completed in month 12. The calculation considers the loss of subjects enrolled during follow-up of 10.0% in the placebo group and 10.0% in the mesoglycan group. 650 subjects will be enrolled and randomized 1:1 in the two placebo and mesoglycan arms.

This number seems large enough to guarantee the study an adequate “power” (> 0.80) even if the risk of observing the primary endpoint in the placebo group (hazard rate control) is the difference in efficacy between the two treatments (hazard ratio) that will deviate significantly from those hypothesized.

However, an “interim-analysis” is prepared when 3/4 of the subjects (n = 488) are enrolled so that the “Data and Safety Monitoring Committee” can reformulate the number of subjects to be enrolled (“resampling”) to reach the preset study power (90%) if the number of primary endpoint events observed up to then deviates significantly from the expected for the two groups, i.e., if the HR differs from the estimated one (50%) exceeded 5% in absolute value (a lower deviation seems tolerable based on the above simulation).

The alpha error is, therefore, conservatively set at 0.025 (O'Brien-Fleming method).

For the interim analysis, a tabular description of the frequency of the primary efficacy endpoint will be provided to the Committee, stratified by treatment group but without its label (i.e., without identifying the nature of the treatment assigned to each group).

### Statistical considerations

All variables will be descriptively analyzed with the appropriate statistical methods: for categorical variables using frequency tables and for continuous variables with sample statistics (e.g., mean, median, standard deviation, minimum and maximum value, 25° and 75° quartile).

Unless otherwise specified, all statistical tests will be 2-tailed and at a 5% significance level.

All the primary and secondary efficacy and safety analyses will be performed in the modified intention-to-treat (ITT) population, including all randomized subjects who received at least one dose of the study drug.

Further supporting analysis will be performed in the per-protocol population, which will include all randomized subjects who were complying with treatment, that is, taking at least 65% of the intended dose units of the study drug.

Subjects who are randomized and who have taken any amount of study drug will not be replaced for any reason. Subjects who will be randomized but withdraw their consent before taking the study drug may be replaced, although they will not lose their unique identifier.

Subjects who leave the observation without manifesting the endpoint will be considered censored, participating in the risk during the observation period. For subjects who do not appear for clinical evaluations at 12 and 24 months of observation, every effort will be made to know a possible outcome of interest for the study, making them evaluable for endpoints.

### Treatment compliance

Compliance with oral treatment can be used as a stratification variable (< 65% and >  = 65%) and defined as compliance = the total number of tablets actually taken / the total number of tablets expected*100.

### Final analysis of the primary endpoints

The primary cumulative efficacy endpoint will be the occurrence of the first event among the event types considered. The hypothesis of superiority of the primary cumulative efficacy endpoint will be tested by multivariate Cox proportional hazards regression using as covariates the assigned drug, age at randomization, sex, compliance with treatment, and the additional risk factors for DVT and DVT/PE found to be significantly associated in the univariate analysis (between obesity, varicose veins, cancer, standing or prolonged immobilization, recent surgery, severe infections, prior DVT/PE, thrombophilia, chronic heart failure NYHA III-IV, use of estrogen-progestogens). All variables participating in the model will be tested for the assumption of proportionality of the risks with the usual graphical method. In addition, a significant subset of variables will be chosen with the Wald method and in a “forward stepwise.”

The crude and adjusted cumulative proportion of event-free subjects over follow-up in the two treatment groups will be described with Kaplan–Meier plots.

### Final analysis of secondary endpoints

For the analysis of the secondary efficacy endpoints, the following will be used:

1. the Kaplan–Meier plot of the cumulative occurrence over time and the log-rank test to compare the two treatment arms,
2. for the rVCSS scores, analysis of variance for repeated measures will be used (all available assessments for within-subject effects and the 2 treatment groups as between-subjects effect).
3. in the case of the VEINES/Sym-Qol scores, the analysis of variance for repeated measures will be used (all evaluations available for the within-subject effect and the 2 treatment groups as the between-subjects effect).
4. The impact of elastic compression therapy in the various endpoints will be analyzed by inserting it as a stratification factor in the related analyzes.
5. The predictive contribution of CRP levels (subdivided by tertiles) to the primary efficacy endpoint will be analyzed by insertion as a stratification factor in relative analyzes.

### Final analysis of the security endpoints

Analysis of the primary safety endpoint will be performed with the continuity adjusted chi-square test.

Standard summary of safety data related to adverse events and significant changes in laboratory data will also be provided.

## Data Availability

The data that support the findings of this study are not openly available due to reasons of sensitivity and are available from the corresponding author upon reasonable request.
